# Prevention and mitigation of congenital toxoplasmosis. Economic costs and benefits in diverse settings

**DOI:** 10.1016/j.fawpar.2019.e00058

**Published:** 2019-06-01

**Authors:** Branko Bobić, Isabelle Villena, Eileen Stillwaggon

**Affiliations:** a Institute for Medical Research, University of Belgrade, Centre of Excellence for Food- and Vector-borne Zoonoses, National Reference Laboratory for Toxoplasmosis, Serbia; b EA 7510, UFR Médecine, University Reims Champagne-Ardenne, National Reference Center on Toxoplasmosis, Hospital Reims, France; c Department of Economics, Gettysburg College, Gettysburg, PA, USA

**Keywords:** CT, congenital toxoplasmosis, PS, pyrimethamine/sulfadiazine, Congenital toxoplasmosis, Prevention, Screening, Economic costs, Economic benefits

## Abstract

Congenital toxoplasmosis (CT), the result of a primary infection of pregnant women with *Toxoplasma gondii* which was transmitted to the fetus, may result in mild to deep injuries occurring in the newborn or later in its development or in adolescence. The visual and cognitive impairment that can result imposes substantial economic costs on the individual and society. Numerous observational studies favor the conclusion that, with preventive measures currently available, it is possible to reduce the incidence of infections in pregnant women, the incidence of fetal infection by preventing transplacental transmission, and the gravity of injury in infected newborns. Treatment of infected newborns can also reduce the severity of consequences and the frequency of their occurrence later in life. Prevention programs, however, are applied in only a few countries; in most countries implementation of a national prevention program has not been considered or has been thought to be too expensive. This article lists the methods of prevention of CT and describes existing national prevention programs in France and Austria. It analyzes the economic costs and benefits of maternal screening for CT prevention and mitigation for society and for health systems. The economic feasibility of implementing national screening in low-prevalence, high-cost countries is illustrated with the example of the United States. New diagnostic tools are discussed and the implication of lower costs is considered, for countries with well-established screening programs as well as those with inadequate prenatal care networks.

## Highlights

• Congenital toxoplasmosis–a significant economic cost to individuals and society
• Prevention reduces incidence, injury severity in newborns, sequelae frequency.
• Benefits and costs of screening programs in France and Austria
• Benefits of screening in low-prevalence high-cost countries–United States example
• Use of new lower-cost diagnostic devices–economic benefits for prevention.

## 1 Introduction

Primary infection with *Toxoplasma gondii* (*T. gondii*) during pregnancy can pose serious risk to the developing fetus that can present in mild to profound lesions that are evident during gestation, at birth, or later in life The consequences of congenital toxoplasmosis (CT) can be prevented or mitigated with early diagnosis and treatment. This article examines the nature of the problem, the methods of prevention, the outcomes of national prevention programs, and the economic costs and benefits of prevention for society and for health systems.

Caused by *T. gondii*, a single celled protozoan parasite, toxoplasmosis is a major zoonosis infecting about 25–30% of the global human population (Montoya and Liesenfeld, 2004). Incidence of CT is estimated to be 190,100 cases globally, equivalent to a burden of 1.20 million Disability-Adjusted Life Years (DALYs) (Torgerson and Mastroiacovo, 2013). The success of *T. gondii* derives from its ability to develop in any type of (nucleated) host cell of all warm-blooded (and many cold-blooded) animal species, including humans, and that it can be transmitted directly from one transit host to another. The infected *Felidae*, as a permanent host, contaminate the environment by feces that contain oocysts. Contaminated environment, including soil, plants, and water, is the infection source for herbivores and birds, and the infection results in the formation of parasitic cysts in their tissues. Consumption of infected meat is the route of infection for carnivores. Humans, as all omnivores, can be infected from all sources: contaminated soil, fruits, vegetables, water, or infected undercooked meat.

In the immunocompetent individual, toxoplasmosis is generally a mild, self-limiting infection. Only about 10% of acutely infected people develop disease, characterized by flu-like symptoms (fever, body aches, fatigue, swollen lymph nodes, headache) (Montoya and Liesenfeld, 2004). Toxoplasmosis can be a serious medical problem, however, in situations where the immune system is underdeveloped (fetus and newborn) or compromised (HIV and other diseases with depression of cellular immunity, or the result of medical treatment).

Infection of the fetus, and thus of the newborn, is the result of vertical transmission of the parasite from the mother. Only acute infection during pregnancy or during the periconceptional period generally leads to CT (Montoya and Liesenfeld, 2004; Peyron et al., 2016; Villena et al., 1998). Rare exceptions have been described in chronically infected immunocompromised pregnant women or in cases of reinfection of pregnant women with highly virulent, generally atypical strains found in South America and Africa (Desmonts et al., 1990; Elbez-Rubinstein et al., 2009). The global incidence of CT is approximately 1.5 cases per 1000 live births (Torgerson and Mastroiacovo, 2013).

The rate of maternal-fetal transmission, without prenatal treatment, is estimated to average 50% over the course of pregnancy (Couvreur et al., 1988; Prusa et al., 2015), and with prenatal treatment is estimated to be 13–30% (Li et al., 2014; Dunn et al., 1999; da Silva et al., 2015). Transmission rate, however, mainly depends, as does the clinical presentation of CT, on gestational maturity at the time of infection (Dunn et al., 1999). Risk of fetal infection is lowest in early pregnancy (< 10%) and highest at the end of the third trimester (60–81%) (Swisher et al., 1994; Dunn et al., 1999; Wallon et al., 2013). On the other hand, when the infection occurs early in pregnancy, the consequences for the fetus will be the most serious, while with gestational maturity the severity of the consequences is reduced. Fetal infections in the first half of pregnancy can result in intrauterine death, hydrocephalus, microcephaly, and seizures (Montoya and Liesenfeld, 2004; Gilbert et al., 2006). While serious consequences may also occur in second- and third-trimester infections, they are less common. Fetal infections are most likely to occur during the third trimester. Clinical signs are often absent at birth, but infected children can develop late sequelae (chorioretinitis or neurological and cognitive disorders) (Montoya and Liesenfeld, 2004; Villena et al., 2010).

Since infection in immune-competent individuals is usually asymptomatic, even in pregnant women (Montoya and Liesenfeld, 2004), clinical diagnosis is rarely established, and even then the lack of specific symptoms makes it unreliable without a laboratory analysis. Immunological tests for the detection of IgG, IgM, IgA, and avidity of IgG-specific antibodies are used for diagnosis of maternal infection, and amniocentesis with molecular analysis is used for the diagnosis of fetal infection. Determining the time of maternal infection is the basis for assessment of the risk of fetal infection and the need for therapy. Current diagnostics can only indicate if the infection of the mother occurred four months or more before the test (based on the avidity of specific IgG antibodies) (Villard et al., 2016; Liesenfeld et al., 2011). If the testing is performed more than four months after the onset of the infection (in advanced pregnancy), precise dating of the infection becomes difficult and unreliable (Štajner et al., 2016). The diagnosis of CT is even more complex. Prenatal and early postnatal diagnosis of CT requires the application of a complex algorithm involving a combination of serological and molecular methods and biological assay (Štajner et al., 2016; Roberts et al., 2001; Pomares and Montoya, 2016). In newborns suspected of infection, a negative finding of any existing laboratory method at birth or in the first six months of a child's life cannot, in itself, exclude intrauterine infection. The recognition of late sequelae is always burdened by differential diagnostic problems (Garweg et al., 2011), and in children without clinical signs at birth the sequelae can occur after several years.

Spiramycin and/or a combination of pyrimethamine/sulphadiazine (PS) are administered to acutely infected pregnant women in order to prevent a fetal infection or its consequences (Desmonts and Couvreur, 1974a, Couvreur et al., 1988; Goldstein et al., 2008; Mandelbrot et al., 2018). The role of spiramycin is to prevent the spread of parasites from the mother to the fetus through the placenta and is introduced after acute infection is suspected (Avelino et al., 2014). The purpose of PS is fetal treatment and is administered to women in whom fetal infection in the child is confirmed or is very probable (Thiébaut et al., 2007). According to a multicenter randomized trial in France, therapy with PS seems to be more effective than spiramycin therapy in the prevention of transmission of infection to the fetus, although the difference did not reach statistical significance (probably due to an insufficient number of pregnant women with seroconversion) (Mandelbrot et al., 2018). Although the effectiveness of the therapeutic approach is not universally accepted (Gilbert et al., 2003), the prevailing judgment is that it is effective and should be administered (Thiébaut et al., 2007). For the treatment to be effective, it is necessary to begin promptly, preferably within 4 weeks of infection (Gras et al., 2005).

## 2 Approaches to prevention and mitigation

Bearing in mind the lack of clinical symptoms, the complexity of diagnosis, and the need for timely application of treatment to prevent serious CT consequences, intervention is carried out at three levels: the prevention of maternal infection during pregnancy, the prevention of transplacental transmission of infection to the fetus, and the mitigation of the consequences of fetal infection.

*Prevention of infection during pregnancy*. Health education to instruct women on how to avoid exposure to possible pathways of infection provides a basic level of prevention for reducing the incidence of primary infection in pregnant women.

*Prevention of transplacental transmission of infection*. Clinical data show that timely treatment of an acutely infected pregnant woman can prevent or delay transmission of the infection to the fetus and, in the event of fetal infection, significantly reduce the frequency or mitigate the severity of consequences (Desmonts and Couvreur, 1974a; Couvreur et al., 1988). The precondition for such a program in any jurisdiction is that the medical society is convinced of the necessity and efficacy of treatment during pregnancy and that curative therapy is officially registered, so it can be easily purchased (El Bissati et al., 2018).

Such a program includes systematic serological screening of pregnant women to identify uninfected women who are at risk of infection and women for whom an acute infection is suspected. Uninfected pregnant women who are at risk of infection are serologically monitored during pregnancy, while women who are suspected of acute infection are treated immediately with spiramycin.

*Mitigation of the consequences of fetal infection*. Mitigation measures are based on the observation that timely treatment of fetuses and/or newborns with PS can alleviate the consequences of infection and reduce the frequency of later consequences. In the event of maternal infection, testing of amniotic fluid is necessary to determine fetal infection and the need for PS. If screening in pregnancy is not implemented, the serological screening of the newborn is a prerequisite for comprehensive mitigation of the consequences of fetal infection (Schmidt et al., 2006). Whether therapy is started before or after birth, it is administered continuously during the first year of life (McLeod et al., 2006).

## 3 Where we are today

CT prevention programs are not applied uniformly worldwide, nor even throughout Europe, which has the longest history of systematic screening. Mandatory serological testing beginning in the first trimester, with the monitoring of seronegative women throughout pregnancy, has been implemented over an extended period in only three European countries: France, Austria and Slovenia. According to reporting for 2016 (ECDC, 2018), screening is applied in Slovakia as well. In Italy, mandatory serological prenatal screening was introduced in 2017 (Regulation, 2017). In recent years, screening has been conducted in Poland as a time-limited national project (personal communication M. Rozycki, 2018). In the Czech Republic, screening programs are in place only in particular regions, and in Belgium, serological control of pregnant women is mandatory in the first trimester but without follow-up of seronegative women (ECDC, 2018). Sweden and Iceland screen only suspected cases or women at high risk on an individual basis (ECDC, 2018). In Germany there is voluntary screening that depends on the initiative of pregnant women themselves or their gynecologists, and it is not always covered by health insurance (ECDC, 2018). Historically, some countries opted for screening of pregnant women or of newborns but have discontinued the programs, as was the case for prenatal screening in Germany (carried out in East Germany until 1991) (Janitschke, 1991), non-systematic prenatal screening in Switzerland (Rudin et al., 2018), and serological screening of newborns in Denmark (1999–2007) (Röser et al., 2010).

Beyond Europe, few countries have programs of systematic serological prenatal screening and follow-up at the national level. Serological control of pregnant women in the first trimester is mandatory in Morocco, but without follow-up of seronegative women (El Bissati et al., 2018). Legislation in 2014 made universal screening mandatory in Panama, but it has not yet been implemented. In Colombia, clinical practice guidelines recommend screening (Cortes et al., 2017), and follow-up of seronegative women is carried out in some parts of the country (Cañón-Franco et al., 2014; El Bissati et al., 2018). Preventive screening has been applied in some regions of Brazil (Lopes-Mori et al., 2011; Avelino et al., 2014).

### 3.1 Health education

There are various models of conducting health education, including the most common oral information provided by healthcare providers, leaflets, lectures, and workshops (Foulon et al., 1994; Breugelmans et al., 2004; Gollub et al., 2008). Brochures issued by reference laboratories or health institutes are available in many countries. In some countries, official documents, such as guidelines for the protection of women's health during pregnancy, prescribe health education by a gynecologist.

The overall effectiveness of prenatal education has not been adequately assessed, nor has the impact of specific educational interventions (Di Mario et al., 2015; Cortes et al., 2017). Prenatal education has a significant impact on the improvement of women's knowledge, but the impact on behavioral changes in women is less certain (results of individual studies analyzed in Di Mario et al., 2015). A long-lasting, continuous campaign is likely to have an impact on behavior in coming generations. Education efficiency can be higher if it is concentrated on a small number of behavioral constraints, but that requires knowledge of the impact of each infection path, which varies between countries and changes over time (Pappas et al., 2009; Bobić et al., 2011). It is important, of course, to educate educators as well (Davis et al., 2015). Using public service messages in the media can extend primary prevention to inform the entire population. A study in California showed that, of the people with prior knowledge about *T. gondii*, 56% found out about toxoplasmosis from newspapers, the internet, books, or public presentations (Dabritz and Conrad, 2010). In Serbia, a similar study showed that, among women with prior knowledge, 40.6% learned about toxoplasmosis through the internet (Bobić et al., 2015). There can be problems, however, with the accuracy of the information offered (Bobić et al., 2015). Correct medical information should be provided on the official site of public health authorities in each country. Education in multinational societies should be multilingual (El Bissati et al., 2018). It is clear that the approach of prevention through health education is applicable in all countries, but it does not solve the problem of pregnant women who become infected in spite of information dissemination.

In a broader sense, the efforts of the veterinary services to improve farm hygiene in order to reduce the prevalence of infection in animals (discussed elsewhere in this issue) can also be classified as primary prevention. The effectiveness of those measures for human health has not yet been adequately determined. A new approach could come with vaccination of cats to reduce the spread of oocysts in the environment (Opsteegh et al., 2015) if a vaccine can be developed.

### 3.2 Serologic screening of pregnant women and fetuses suspected of infection

France, Austria, and Slovenia have the longest experience with systematic programs of universal screening in pregnancy. The monitoring of seronegative pregnant women according to the French national program is scheduled monthly, and in the Austrian and Slovene programs, screening during pregnancy occurs at 8-week intervals.

#### 3.2.1 French national program of prevention of congenital toxoplasmosis

France has moderate prevalence of toxoplasmosis among women of childbearing age, with a substantial decrease in recent years from about 40% in 2003 to 31% in 2016. Incidence of seroconversion in pregnant women was estimated through modeling to be 2.5 (95% CI 1.9–3.2) per 1000 susceptible women in 2010. Long-term predictions suggest that prevalence will continue to decline to 18%, 27%, and 38% for women aged 20, 30, and 40 years respectively by 2020 (Nogareda et al., 2014). The national prevention program for CT in France has been in place since 1978 and consists of screening women in the first trimester of pregnancy for a program of education and surveillance. In the case of seronegative women, serological surveillance continues monthly until delivery. In the event of seroconversion, the recommended protocol includes treatment with spiramycin and prenatal diagnosis by amniocentesis with fetal ultrasound monthly. In the case of positive prenatal diagnosis, treatment by PS combination with folinic acid is administered to reduce long-term sequelae. Experience with this protocol has demonstrated the effectiveness of treatment for recently infected pregnant women in reducing rates of vertical transmission and severity of congenital toxoplasmosis (Wallon et al., 2013). Abortion is not considered warranted except for a fetus with severe lesions identified by ultrasound (such as hydrocephalus, microcephaly, or disseminated infection). At birth, infants with congenital toxoplasmosis receive additional testing, including cranial ultrasound, funduscopy, and complete blood count to monitor treatment, and are treated for one year.

Until 2007, no data were available concerning the annual number of cases of congenital toxoplasmosis, nor the severity of cases of CT. In order to estimate the perinatal burden of this infection and to monitor the impact of the national prevention program, a surveillance system based on declaration of cases was implemented in 2007 by the National Reference Centre for Toxoplasmosis (NRC) in collaboration with Santé publique France (http://cnrtoxoplasmose.chu-reims.fr). The NRC is responsible for this system, with the collaboration of specialized and medical biology laboratories (King et al., 2008).

Cases of CT diagnosed in France (in antenatal or postnatal period) are reported to the NRC for Toxoplasmosis each year using specifically developed software, Voozanoo (EpiConcept, Paris, France). A case of CT is defined as a fetus, newborn, or infant aged less than one year with at least one of the following:

• *T. gondii* in body tissues or fluids (amniotic fluid, neonate blood) identified by polymerase chain reaction (PCR), inoculation of mice, or cell culture (these two last methods are no longer used for diagnosis),
• specific IgM or IgA antibodies at birth confirmed within 10 days of life,
• specific IgG antibodies within the first 12 months of life,
• persistent IgG positivity until one year of age.

The system aims to collect information on cases of CT diagnosed during pregnancy by amniocentesis, or diagnosed in newborns and infants less than one year whose mother had seroconverted during pregnancy. Patient data are reported, such as estimated gestational age at the time of maternal infection and age of mother), pregnancy outcome (abortion, fetal death, or living newborn), and clinical status of the newborn or child (particularly neurological lesions and visual impairments, e.g. chorioretinitis, with localization) (King et al., 2008).

The data obtained by this surveillance are collected by NRC in Reims and used to produce an annual report destined for relevant health professionals. The objectives of the surveillance are to estimate overall prevalence of the disease in France, monitor prevalence trends, and estimate the proportion of cases with severe forms of infection (hydrocephalus, microcephalus, and macular chorioretinitis).

Since the beginning of this surveillance, 2276 cases of CT were reported for the period 2007–2016, with antenatal diagnosis in 899 cases and postnatal diagnosis in 1377 cases. Amniocentesis was performed in 1008 cases and 899 were positive; 2025 infected children were born; some pregnancies were not followed up. Of the congenitally infected children, 90% of them were diagnosed before the age of 2 months. Western blot (performed in specialized laboratories) was used for 52% of diagnoses, and the presence of serum IgM/IgA of infants aged 0–2 months contributed 87% of diagnoses. Thus, with brief delay, children could benefit from early treatment (PS), with the expectation of a favorable outcome. Late diagnosis was generally due to inadequate follow-up of children and absence of regular serology recommended by the NRC and the French National Authority for Health (HAS).

A total of 105 terminations of pregnancy were reported: 76 abortions were performed for medical reasons (cerebral lesions were detected by ultrasound examination in four cases) and there were 29 fetal deaths. No pregnancies were terminated following late maternal infection. Among 2025 live-born infants, 1836 (90%) were without signs of disease and 189 (10%) had visible lesions; 129 had moderate lesions (intracranial calcifications and/or peripheral chorioretinitis) and 60 (31%) had a severe form of the disease (hydrocephalus and/or macular chorioretinitis).

In 2007, the overall prevalence of CT observed was 3.3 (95% CI: 2.9–3.7) per 10,000 live births, and the estimated incidence rate of CT with lesions was 0.34 (95% CI: 0.2–0.5) per 10,000 live births. Mean prevalence of CT over the period 2007–2016 was 2.8 per 10,000 live births.

The number of cases directly observed by this surveillance is probably more reliable than that previously reported from different estimations; this is due to the exhaustive process adopted for notification (all laboratories carrying out the diagnosis in France were invited to participate in the surveillance). The low rate of severe forms of CT observed in France is probably due to the prevention program in practice since 1978.

There is a limitation to this surveillance, only the lesions observed in newborns and infants during the first few months will be detected and reported. The true burden of CT should be evaluated by long-term follow-up of cases because congenitally infected newborns without signs of CT at birth are still at risk of developing ocular lesions during childhood and adolescence, leading to visual impairment (Montoya and Liesenfeld, 2004). Long-term case follow-up, however, was not the objective of this surveillance program. It was intended to help health professionals monitor tendencies of infection in France, in the event that CT prevention policy would change in coming years. The screening program is free of charge for families; costs are covered by the national health insurance funds.

#### 3.2.2 Austrian national program of prevention of congenital toxoplasmosis

The Austrian national program of prenatal screening and treatment to prevent or mitigate the effects of CT provides a complementary perspective to that of France. Austria is considered to have moderate prevalence of toxoplasmosis among women of childbearing age, about 33% (Prusa et al., 2013; Sagel et al., 2011; Edelhofer and Prossinger, 2010). That was not always the case. A study by Thalhammer in 1961 found a rate of CT of 78 per 10,000 live births in Austria (Thalhammer, 1961). To respond to the high levels of CT and consequent injuries in newborns and youth, the government initiated a program of mandatory prenatal screening for *T. gondii* infection for all pregnant women that began in 1974 under the auspices of the national health care system (Thalhammer, 1961; Thalhammer and Heller-Szollosy, 1979). Prenatal care, including such screening, is part of a national prevention program called “Mother-Child-Booklet” program for all pregnant women and their infants through early childhood. The program is free of charge for families; costs are covered by the government and the health insurance funds in each region.

Previous works describe the Austrian national program in detail (Prusa et al., 2013; Prusa et al., 2015). Serological prenatal screening begins ideally at 8 weeks. Women with proven seropositivity before the current pregnancy are not tested. Women who are tested at 8 weeks and found to have been seropositive before conception require only that test. Seronegative women are retested on a bimonthly schedule, at 16, 24, and 32 weeks of gestation. In the case of women seronegative up to the time of delivery and women who have not been tested during pregnancy, cord blood is tested (Prusa et al., 2013). Women with suspected primary infection during pregnancy are tested twice in regional laboratories, and seroconversions and ambiguous results are retested in the reference toxoplasmosis laboratory at the Medical University of Vienna. About 60% of women with primary infection had amniocentesis and polymerase chain reaction testing of the amniotic fluid, the costs of which are not covered by the national program but are absorbed by the Medical University toxoplasmosis unit (Prusa et al., 2015).

In the years 1992 to 2008, 93% of pregnant women had some screening, although most women did not have the prescribed number of tests (Prusa et al., 2013). Women diagnosed with primary infection during pregnancy are treated until delivery and infants with proven or suspected congenital infection are treated for the first year of life. Infants with CT receive additional testing, including cranial ultrasound and funduscopy, and complete blood count to monitor treatment, beyond the standard well baby care.

Austria's national program of CT prevention has been meticulously documented, with the serology history and birth outcomes for 1,387,680 pregnant women from 1992 to 2008 recorded in the Austrian Toxoplasmosis Register (Prusa et al., 2015). The Register reported 10% of women with proven seropositivity before pregnancy and an additional 24.4% of women whose immunity was confirmed through screening (Prusa et al., 2013). Without the national screening program the mother-to-child transmission rate was 50.8% and cases of CT were almost 1% of births. With screening and treatment, in the 17 years from 1992 to 2008, with 77,000 births per year, the Register documented 70 women with primary infection of *T. gondii* per year and 8 cases of CT per year (about 1 per 10,000 births). Records of long-term follow-up to 2013 revealed that 81% of infants with *T. gondii* infection showed no clinical signs, and no children had profound injuries. Some of the small number with visual or cognitive impairment required special school programs, but no children were unable to finish school or enter the workforce (Prusa et al., 2015).

The Austrian national program is successful in CT prevention and mitigation in a number of ways. As a systematic program of regular monitoring, it has the corollary effect of educating the population about food safety, especially during pregnancy, reducing incidence of maternal infection. By treating mothers early in pregnancy, it has reduced mother-to-child transmission from 51% to 11% (that 11% includes mothers who were not screened). Moreover, the continued treatment of mothers over the course of the pregnancy and treating infants has reduced the number of children with signs of CT and reduced the severity of lesions in children with CT. The continued surveillance at birth allows the treatment of infants not otherwise detected, preventing or mitigating lesions in infancy or childhood. Ultimately, over the period recorded in the register, cases of CT decreased from 78 per 10,000 to 1 per 10,000, with few serious lesions in infected children (Prusa et al., 2015).

The countries with systematic prenatal screening and treatment programs face the paradox of successful prevention. Now there are so few children with serious, disabling consequences of CT that it can appear that the risk of CT does not warrant spending for universal prenatal screening programs. In Austria, the government undertook a major overhaul of the Mother-Child-Booklet program, which threatened to end funding for toxoplasmosis screening because children with serious injuries from CT were not seen anymore. It was, of course, because of the success of the screening program that so many children were saved from the mild to profound injuries of CT. *T. gondii* is still present in the food supply, women are still at risk, and babies are still at risk. In reality, the success of education programs has meant that prevalence among women of childbearing age is declining, leaving more women non-immune and at risk during pregnancy.

Only one other European country, Slovenia, has a national program of prenatal screening and maternal and infant treatment to prevent or mitigate the effects of CT. The Slovene program, mandatory since 1995, is structured the same as that of Austria, with bimonthly serology in seronegative women. The Slovene program has had outstanding success and there are no children at present with injuries of CT and only one child under observation whose mother was first screened late in pregnancy (personal communication, M. Skvarč, Ljubljana, Slovenia, 2017).

## 4 Economic costs and benefits of prevention programs

### 4.1 Health education

We have already pointed out that the efficiency of prenatal education has not been precisely evaluated, although as part of the preventive programs of Austria and France, it has apparently been effective. As with other public health interventions, the benefits of education are diffuse, have spillover benefits, and are impossible to capture fully in a cost-benefit model. Advice on how to avoid *T. gondii* infection can also have an impact in the prevention of most other food-borne infections. Moreover, with any public health intervention, other ongoing influences, such as the opinions of family, friends, and the media, or environmental changes that affect soil or water, confound efforts at precise measurement. Given the small cost of basic implementation of education in the context of ongoing prenatal care (costs of brochures, doctors' time to give advice, placing guidance on the official government website), providing women with the information they need to protect their infants is both ethical and practical.

### 4.2 Economic analysis of national toxoplasmosis screening programs

#### 4.2.1 Economic analysis of the French program

Thus far, there is no published economic analysis of the French national program of screening and treatment. In her doctoral thesis in 2003, Binquet established the thresholds for the greater cost-effectiveness of prenatal screening compared to screening at birth to prevent injuries from CT, measured in the short term (infancy) and at 15 years of age. The key parameters influencing cost-effectiveness in Binquet's study were the incidence of primary infections of toxoplasmosis during pregnancy and the treatment effect (reduction in maternofetal transmission) (Binquet, 2003). Considering only the number of cases of CT avoided, not the gravity of injuries in untreated children, Binquet demonstrated that maternal incidence of 0.4% coupled with a reduction of maternofetal transmission of 81% were sufficient to establish the greater cost-effectiveness of prenatal screening (Binquet, 2003). When considering all the adverse events at 1 year and up to 15 years (sequelae, neonatal deaths, fetal losses, as well as termination) and a 0.4% incidence rate, prenatal screening would be considered cost-effective if prenatal treatment reduced the risk of maternofetal transmission by as little as 9% (Binquet, 2003).

The French program has apparently been successful in educating pregnant women to avoid sources of infection, thus reducing incidence of seroconversion. The modeling mentioned in Section 3.2.1 estimated 0.25% incidence of seroconversion (25 per 10,000 susceptible women) in 2010 (Nogareda et al., 2014). NRC surveillance for 2007 to 2016 found 2.8 cases of CT per 10,000 live births (0.028%). If the maternal seroconversion estimate is correct, then maternofetal transmission was about 11.2%. Historical estimates of maternal transmission without prenatal treatment were 50% over the course of pregnancy (Couvreur and Desmonts, 1962; Remington et al., 2010; Desmonts and Couvreur, 1984; Desmonts and Couvreur, 1974b; Forestier, 1991; Stagno et al., 1977). That is the same rate found in Austria more recently in untreated mothers (50.8%) (Prusa et al., 2015). That would suggest a 78% decrease in maternofetal transmission (prenatal treatment effect), certainly within the range of Binquet's (2003) estimate of the threshold for cost-effectiveness.

Binquet's analysis, moreover, did not measure the gravity of injuries in children, only the cases of CT and the occurrence of any undesirable event (Binquet, 2003). The outcomes of interest, however, are not simply binary. It is essential to recognize the profound effect of treatment on the rate of severe CT and the gravity of lesions in affected children in the NRC results. Only 10% of infants with CT (of treated mothers) had lesions, of whom 31% had severe injuries. These results, however, are short term and do not include ocular or other problems that present in the developing child or adolescent. Those outcomes widen the gap between prenatal and post-natal treatment in favor of screening. The reduction in CT with lesions and in the gravity of lesions substantially reinforces Binquet's (2003) finding regarding the cost advantages of prenatal screening.

#### 4.2.2 Economic analysis of the Austrian program

The Austrian government undertook a reevaluation of all services in the Mother-Child-Booklet, requiring justification for each program. Given the government challenge to the toxoplasmosis program, it was necessary to demonstrate that screening and treatment were not only efficacious in reducing and mitigating CT, but that the program was also cost saving. A benefit-cost analysis of the Austrian program demonstrated that it is an outstanding investment for maternal and child health. The analysis calculated costs from the societal perspective, that is, including direct costs of lifetime care and accommodation for affected children plus the lost productivity and loss of life that would have occurred if Austria had not implemented the screening program. That counterfactual was compared to the actual costs of screening, treatment, and lifetime care for children under the screening program. The analysis used clinical data from the Austrian Toxoplasmosis Register and actual costs for testing and treatment in Austria during the period (Prusa et al., 2017).

The analysis found that the screening and treatment program saved €448 million over 17 years, about €26 million per year. The screening program costs €1.9 million per year. The societal benefits of screening are 14 times the program cost, an excellent investment for Austrian society (Prusa et al., 2017).

The benefits and costs of a social investment are generally evaluated from the societal perspective, as described above. In the event, however, that the objections to a program are from the more narrow perspective of a government budget, it is possible to compare the savings in budgetary terms to the costs of a program. The Austrian study also calculated the impact on just the Austrian public budget—that is, omitting the lifetime costs of lost earnings that fall on affected children, their families, and society, and the value of fetal and infant deaths—and found that the maternal screening program is still cost-saving. Strictly budgetary savings were more than €258 million, more than €15 million per year. Thus the Austrian government savings per year were 8 times the annual cost of the prenatal screening and treatment program (Prusa et al., 2017).

It is useful to see these costs in relation to overall Austrian government spending and Gross Domestic Product (GDP), which measures national output. The annual cost of the toxoplasmosis screening and treatment program is 0.007% of total Austrian public spending on health and 0.003% of overall Austrian government spending. The annual cost of the program is 0.0006% of Austrian GDP (Prusa et al., 2017).

The Austrian national program of prenatal screening has had outstanding success in reducing incidence of CT (Berghold et al., 2016) and eliminating or mitigating the injuries that afflict infected infants and children. It has also been demonstrated that saving the lives of affected children or protecting their sight and cognitive function offers very large cost savings to society and to the government budget. The oft-stated presumption that preventive programs are not cost-saving is incorrect because the actual costs of such programs are trivial in comparison to the long-term effects of congenital injuries, the costs of which are overlooked in simple comparisons.

An interesting finding of the Austrian program was that its success compares favorably with that of France although the Austrian program is based on testing every 8 weeks. Moreover, in Austria only a small percentage of women had the suggested number of blood tests (Prusa et al., 2017). These findings merit further scrutiny in a cross-country comparison. Both the French and Austrian experience, however, demonstrate that a robust national program can have enormous impact on child survival and wellness, in spite of the fact that compliance may never be perfect.

#### 4.2.3 Economic evaluation of the costs and benefits of initiating a maternal screening program in a low-prevalence, high-cost setting

A long-standing debate in health economics has centered on the efficiency of broad interventions in the context of low prevalence (Walsh and Warren, 1979; Rose, 1985; Ahern et al., 2008). Economic analyses that consider lifetime costs of disabling diseases, however, consistently show that prevention can be cost-saving, even in low-prevalence settings (Stillwaggon et al., 2018). The low costs of screening and the high costs of neglect are the motivating factors for numerous screening tests during pregnancy. Toxoplasmosis screening is a good candidate for inclusion in routine pregnancy testing even in low-prevalence settings.

In the United States, national prevalence of toxoplasmosis is about 11% for women of childbearing age (https://www.cdc.gov/parasites/toxoplasmosis/epi.html). The United States has no national program of systematic prenatal or postnatal screening for toxoplasmosis. Very few cases of CT are identified prenatally. Without education, mothers giving birth to congenitally infected infants in the United States commonly do not recognize risk factors for which education would have been effective (Boyer et al., 2005). Without systematic screening, development of lesions is uninterrupted. A recent study of children in the United States with CT who had no pre- or postnatal treatment found that 91% of the children who were referred had visual and/or mental impairment by 12 years of age (Olariu et al., 2011).

In 2011, an economic model was developed for the United States with a hypothetical program of monthly testing according to the French protocol and using US prevalence, incidence, and costs for testing, treatment, and lifetime accommodation for children with toxoplasmosis injuries (Stillwaggon et al., 2011). From several studies in the United States, an estimate was derived for incidence of primary infection in pregnancy of 0.11% (11 per 10,000 susceptible women). The US study demonstrated that even in a low-prevalence, high-cost setting, such as the United States, the benefits far outweigh the costs of a national program, even at the high testing costs of 2011. The analysis demonstrated that a maternal screening program in the United States would save almost $2.5 billion per year due to high lifetime costs of caring for and educating children with visual and cognitive impairments caused by CT. The results were robust to differences in maternal prevalence and incidence, and screening was found to be cost-saving for incidence of CT as low as 0.01% (1 per 10,000 births) (Stillwaggon et al., 2011).

The US study used a cost for serology ($12) that was standard in some settings in the United States. A challenge with any cost study in the United States is that there can be enormous variations in prices that labs charge, from hospital labs to commercial labs used by doctors' offices, with no correspondence between the cost of producing the service and the charge for the service. In the absence of a single payer system or regulation of pharmaceutical, laboratory, and provider services, cost-to-charge ratios vary greatly from state to state and among types of providers. (For extensive discussion of cost-to-charge ratios in the United States, see Stillwaggon et al., 2018, methodological supplement). The variation in serology costs raises concerns for a US national screening program (Maldonado et al., 2017).

## 5 Opportunities for expanding prevention programs

Comprehensive health education for prevention of CT is warranted in all countries, with each country determining the selection of content that would be most effective in the context of its population and environmental factors. The best results, however, can be expected if health education is part of a more comprehensive screening program for pregnant women and potentially infected fetuses.

The development of new diagnostic tools makes screening less expensive and offers opportunities for exploiting economies of scale and scope in prenatal testing. One innovation is point-of-care (POC) testing, which has been shown to be reliable in a variety of settings (Begeman et al., 2017; Lykins et al., 2018; Peyron and McLeod, 2018), potentially reducing the costs for established programs in Europe and making national programs even more economically beneficial in the United States and other low-prevalence settings. In Europe, national health systems could have lower costs for universal screening, even though serology costs are already predictable.

In France as seroprevalence is decreasing, the population of seronegative (non-immune) women to monitor during pregnancy is greater. That entails a modest increase in costs for screening for CT. Since women are already consuming prenatal care services throughout pregnancy, the marginal cost of toxoplasmosis serology, even for a larger non-immune population, is a very small part of a national prenatal care budget. Nevertheless, a decrease in the cost of this screening program would help to assure the continuation of the screening, with its important impact on child welfare and the reduction of lifetime costs of injuries. In this context, the novel POC test, the *Toxoplasma* ICT IgG-IgM test (LDBIO Diagnostic, Lyon, France; LDBIO), is already approved for clinical use and commercially available in France. It has been found to have very good diagnostic performance when tested for detection of *Toxoplasma* infections from *T*. *gondii* strains circulating in France, with 97% sensitivity and 96% specificity, and it detected seroconversion in pregnant women (Chapey et al., 2017). Moreover despite its higher cost compared to Elisa tests, implementing such a test in France for screening could be attempted, but the test needs to be evaluated on a larger scale. This test is, however, just a screening test and patients should be informed that confirmatory testing will be necessary for a definitive result. Another path for economizing could be to use only a test measuring the total IgG (and not the two isotypes, IgG and IgM) in sera in screening, and in case of negativity, these measures could be repeated. To minimize the cost of screening, POC testing for *T. gondii* based on testing saliva would reduce the need for venipuncture, but this practice would have to be evaluated in the context of serological screening and for diagnosis of seroconversion in pregnant women. In countries with moderate prevalence, screening prevention programs, such as in France, should be continued to detect seroconversion followed by prenatal diagnosis in amniotic fluid in the event of maternal infection.

In the United States, POC testing could be especially important in reducing costs for personnel, equipment, and delivery to labs. Women are already tested for numerous conditions during pregnancy in the United States. Simplifying testing and reducing costs make it possible introduce systematic screening for multiple maternal conditions, such as toxoplasmosis and Chagas' disease, along with HIV, syphilis, and other infections with congenital sequelae that are already standard in the panel of prenatal testing. At a minimum, in low-prevalence settings the low-cost test would be useful for screening at the beginning of pregnancy to detect seronegative women and give them the hygienic and dietary advice to avoid *Toxoplasma* infection.

In countries with less developed prenatal care networks and fewer resources, the benefits of an inexpensive POC test with excellent predictive value are even more important, including lower cost, better coverage, and faster diagnosis and treatment, reducing infections and injuries in neonates and children. Panama, for example, made toxoplasmosis testing mandatory in 2014, but implementation is limited. Point-of-care testing propels this initiative forward and creates a platform for enhancing prenatal care on a national scale. The POC test, at a probable cost of US$4, administered under a nationally mandated protocol of monthly prenatal visits can be an entry point for surveillance for multiple risks to the mother and child. Even in simple clinical settings in remote areas, the prenatal visit can include a blood pressure check, a glucose test, and a conversation with a nurse with basic training. The additional cost of adding another service, while developing a prenatal care platform, is trivial and is referred to as economies of scope (Begeman et al., 2017).

In countries with developed prenatal care and maternal and newborn screening programs, a future improvement can be the long-term follow-up of infected newborns to the third decade of life. Among children with CT who were treated prenatally and postnatally for the first year, of those who developed chorioretinal lesions up to age 14 (24%), 40% developed lesions between the ages of 2 and 10 years (Binquet et al., 2003). In a different study, 41% of children who were treated only postnatally developed lesions from the age of 10 years and older (Phan et al., 2008). That signals the need for long-term clinical follow-up. Follow-up of infected newborns with intracranial calcification or other non-ocular manifestations at birth is particularly important because it appears that the occurrence of chorioretinitis is more common in these children (Binquet et al., 2003; Kieffer et al., 2008).

## 6 Conclusion

Several avenues exist to reduce primary infection with *T. gondii* in pregnancy, including comprehensive education in prenatal visits, public service advertisements, and expanded use of the internet by government ministries. Moreover, experience in France, Austria, and Slovenia demonstrates that maternal screening is effective in reducing CT and reducing or mitigating the consequences of CT in affected newborns and children. Cost-benefit analyses have also demonstrated that such prevention is cost saving from a societal and budgetary perspective.

Economic analyses of health interventions, particularly for prevention, have tended to underestimate the benefits and overstate the costs. A short-term perspective fails to recognize the lifetime costs to the individual and the community due to prenatal and childhood injuries. The productivity losses from preventable, sometimes profound, injuries are staggering. The presumption that screening and prevention are expensive does not withstand careful examination because the lifetime costs of injury are great and the costs of screening trivial in comparison. Given the evidence of efficacy that is available, the decision to spend resources on prevention is a political choice that reflects the priorities of the society.

## Declaration of Competing Interest

The authors declare they have no conflicts of interest whatsoever.
